# Aged mice are less susceptible to motion sickness and show decreased efferent vestibular activity compared to young adults

**DOI:** 10.1002/brb3.3064

**Published:** 2023-07-03

**Authors:** David Lorincz, Hannah R. Drury, Doug W. Smith, Rebecca Lim, Alan M. Brichta

**Affiliations:** ^1^ School of Biomedical Sciences and Pharmacy The University of Newcastle Callaghan New South Wales Australia

**Keywords:** aging, c‐Fos, motion sickness, vestibular efferent

## Abstract

**Introduction:**

The efferent vestibular system (EVS) is a feedback circuit thought to modulate vestibular afferent activity by inhibiting type II hair cells and exciting calyx‐bearing afferents in the peripheral vestibular organs. In a previous study, we suggested EVS activity may contribute to the effects of motion sickness. To determine an association between motion sickness and EVS activity, we examined the effects of provocative motion (PM) on c‐Fos expression in brainstem efferent vestibular nucleus (EVN) neurons that are the source of efferent innervation in the peripheral vestibular organs.

**Methods:**

c‐Fos is an immediate early gene product expressed in stimulated neurons and is a well‐established marker of neuronal activation. To study the effects of PM, young adult C57/BL6 wild‐type (WT), aged WT, and young adult transgenic *Chat‐gCaMP6_f_* mice were exposed to PM, and tail temperature (*T*_tail_) was monitored using infrared imaging. After PM, we used immunohistochemistry to label EVN neurons to determine any changes in c‐Fos expression. All tissue was imaged using laser scanning confocal microscopy.

**Results:**

Infrared recording of *T*_tail_ during PM indicated that young adult WT and transgenic mice displayed a typical motion sickness response (tail warming), but not in aged WT mice. Similarly, brainstem EVN neurons showed increased expression of c‐Fos protein after PM in young adult WT and transgenic mice but not in aged cohorts.

**Conclusion:**

We present evidence that motion sickness symptoms and increased activation of EVN neurons occur in young adult WT and transgenic mice in response to PM. In contrast, aged WT mice showed no signs of motion sickness and no change in c‐Fos expression when exposed to the same provocative stimulus.

## Graphical abstract

In contrast to two strains of young adult mice, which exhibited typical motion sickness responses to provocative motion, aged wild‐type mice displayed no symptoms. Furthermore, efferent vestibular neurons showed increased c‐Fos expression following provocative motion in young adult, but not in aged mice.

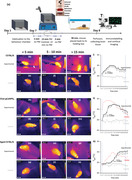

## 1 INTRODUCTION

Motion sickness often occurs when there is a difference between actual and expected motion and is thought to result from discrepancies between the senses of balance (vestibular), proprioception, and vision (Golding & Gresty, 2005, 2015; Oman, 1990; Reason & Brand, 1975). This “sensory conflict” theory was first proposed by Reason and Brand (1975); however, the exact neurobiological mechanism that allows sensory conflict to trigger motion sickness symptoms such as dizziness, sweating, excessive saliva production, nausea, and emesis is still unclear. The vestibular system appears to be a key component in the development of motion sickness. Symptoms of motion sickness are abolished in rats (Morita et al., 1988), monkeys (Wilpizeski et al., 1987), and dogs (Money & Friedberg, 1964) that have had their vestibular organs removed (Money, 1970).

Susceptibility to motion sickness also appears to be age dependent. Older humans (>50 years) are significantly less vulnerable to motion sickness than younger age groups, although infants and young children below 2 years old are thought to be invulnerable (Takeda et al., 2001). Therefore, age‐related changes in motion sickness susceptibility are likely related to the well‐documented deterioration in vestibular function in humans and reported in other animals including laboratory rats (McCaffrey & Graham, 1980; Morita et al., 1988) and mice (Tung et al., 2014). Documented vestibular deterioration includes loss of hair cells and associated nerve fibers, thus attenuating vestibular afferent input to brain centers involved in the coordination of movement (Bergström, 1973a, 1973b; Ji & Zhai, 2018; Rosenhall & Rubin, 1975; Zalewski, 2015). While reduction of motion sickness susceptibility could be regarded as a positive outcome of vestibular senescence, it should be remembered other outcomes such as imbalance and falls are major causes of injury in the elderly (Iwasaki & Yamasoba, 2015; Sloane et al., 1989).

In this study, we focused on the enigmatic feedback component of the vestibular system, the efferent vestibular system (EVS), which is a much less studied area in relation to motion sickness and aging. The EVS originates bilaterally from the brainstem efferent vestibular nucleus (EVN) and provides rich, mainly cholinergic innervation to vestibular hair cells and afferent nerve terminals and parent fibers (Holt et al., 2011; Mathews et al., 2017; Poppi et al., 2020). The precise function of the EVS is still unclear, but evidence suggests the mammalian EVS has mixed effects, exciting primary vestibular afferents, and afferent calyx terminals in the periphery (Schneider et al., 2021) while simultaneously inhibiting type II hair cells (one of two hair cell types in the vestibular neuroepithelium) through the activation of alpha‐9 nicotinic acetylcholine receptors and SK (small conductance) potassium channels (Poppi et al., 2018). Thus, the EVS has a surprisingly complex effect on the afferent sensory output from the vestibular organs of the inner ear. Previous results from our laboratory also suggest the involvement of EVS in motion sickness. Mice with an attenuated EVS, that is, alpha‐9 nicotinic receptor *knockout*s, displayed reduced motion sickness symptoms (Tu et al., 2017).

A characteristic and obvious sign of motion sickness is emesis, which occurs in humans and other mammalian species such as monkeys (Ordy & Brizzee, 1980), dogs (Benchaoui et al., 2007), and shrews (Horn et al., 2014). One notable exception, however, is rodents such as rats and mice, which are unable to vomit. Therefore, to determine if these common laboratory animals experience motion sickness, other indicators are used. These include pica, the consumption of nonnutritious substances (Mitchell et al., 1977; Morita et al., 1988), increased defecation (Ossenkopp & Frisken, 1982), suppression of drinking (Haroutunian et al., 1976), and thermal responses such as transient increasing tail temperature (*T*_tail_) and decreasing core body temperature (Del Vecchio et al., 2014; Guimaraes et al., 2015; Ngampramuan et al., 2014). It has been previously reported by our group (Tu et al., 2017) and others (Rahman & Luebke, 2022) that these thermal responses are a robust and accurate indicator of motion sickness in laboratory mice and can be recorded simply using infrared imaging. We used this method to monitor the response to provocative motion (PM) in different mouse strains and an aged cohort.

The detection of c‐Fos protein is regarded as a reliable marker for the study of neuronal activation following a behavioral stimulus. c‐Fos is an early‐gene product produced by neurons after increased action potential discharge. The protein is generally concentrated in the cell nucleus and its production peaks approximately 90 min after an adequate stimulus (Hoffman et al., 1993; Kovács, 2008; Miller & Ruggiero, 1994; Zhu et al., 1995). Using fluorescent immunolabeling and confocal imaging, *cfos* mRNA and c‐Fos protein expression can be precisely localized in activated cells. Despite its extensive use, c‐Fos labeling, however, has a significant temporal resolution limitation. Due to its extended time‐to‐peak, using c‐Fos expression to identify the exact time course of neuronal activation is challenging. Therefore, c‐Fos detection is often better suited to identifying the involvement of brain regions and cell groups in the neurobiological processing of an applied stimulus (Kovács, 1998). Similar to a study of c‐Fos expression in rat medial vestibular nucleus (MVN) after sinusoidal galvanic stimulation of the vestibular periphery (Holstein et al., 2012), we used c‐Fos expression to study possible activity changes of the murine EVN after provocative horizontal orbital motion in young adult and aged mice.

## 2 MATERIALS AND METHODS

### 2.1 Experimental animals and ethical statement

All experimental procedures were approved by the University of Newcastle Animal Care and Ethics Committee prior to experiments (ethics approval number: A‐2020‐025). Mice were housed at the University of Newcastle under the same standard conditions with a 12‐h dark/light cycle, constant humidity and temperature (60%; ∼22°C), and water and food available ad libitum. Wild‐type (WT) “C57BL/6 WT” (C57BL/6J, JAX #000664) is one of the most widely used inbred laboratory mouse strains and the foundation strain of numerous transgenic lines. Two age groups of C57BL/6 mice were used in our study: young adults (4–10 months) and “aged” (>24 months). In addition, we used transgenic “Chat‐gCaMP6f” heterozygous mice, which were created crossing homozygous Chat‐Cre mice (B6;129S6‐Chattm2(cre)Lowl/MwarJ; JAX #028861) with homozygous Ai95D mice (B6J.Cg‐Gt(ROSA)26Sortm95.1(CAG‐GCaMP6f)Hze/MwarJ; JAX #028865). Chat‐gCaMP6f crossbred mice express the genetically encoded calcium indicator protein gCaMP6_f_ in all cholinergic neurons including the EVN. We used Chat‐gCaMP6f strain to determine if transgenic mice behaved the same as WT mice and because this strain has the potential to be used for calcium imaging studies. Mice from all three mouse cohorts (young adult C57BL/6 WT, *Chat‐gCaMP6_f_*, and aged C57BL/6 WT) were randomly divided into *control* and *experimental* groups. All control and experimental groups underwent *T*_tail_ recordings and c‐Fos immunolabeling, but only the experimental groups were exposed to PM.

Genotyping was done in conjunction with Australian BioResources (Moss Vale, New South Wales) and the Garvan Institute (Sydney) using standard forward and reverse primer sequences recommended by Jackson Laboratory for these commercially available transgenic strains.

### 2.2 PM and *T*_tail_ recordings

Mice used in these experiments were as follows: C57BL/6 WT (control: *n* = 3, female; PM: *n* = 14, female), aged‐C57BL/6 WT (control: *n* = 3, male; PM: *n* = 5, male), and *Chat‐gCaMP6_f_* (control: *n* = 2, female; PM: *n* = 5, 1 male and 4 female).

A recording chamber (40 × 25 × 30 cm [*l* × *w* × *h*]) with elevated red walls was placed on a laboratory orbital shaker (Model E0M6, Ratek, Australia), and a suspended infrared camera (FLIR‐E50, Flir Systems, Wilsonville, OR, USA) was used for monitoring *T*_tail_ by taking infrared images of the mouse from above.

All mice were habituated to the setup for 7 days prior to recordings to minimize stress induced by handling and exposure to a new environment, which can potentially mask elevated *T*_tail_ response to PM. The chamber was thoroughly cleaned with 70% ethanol after each mouse.

**FIGURE 1:**
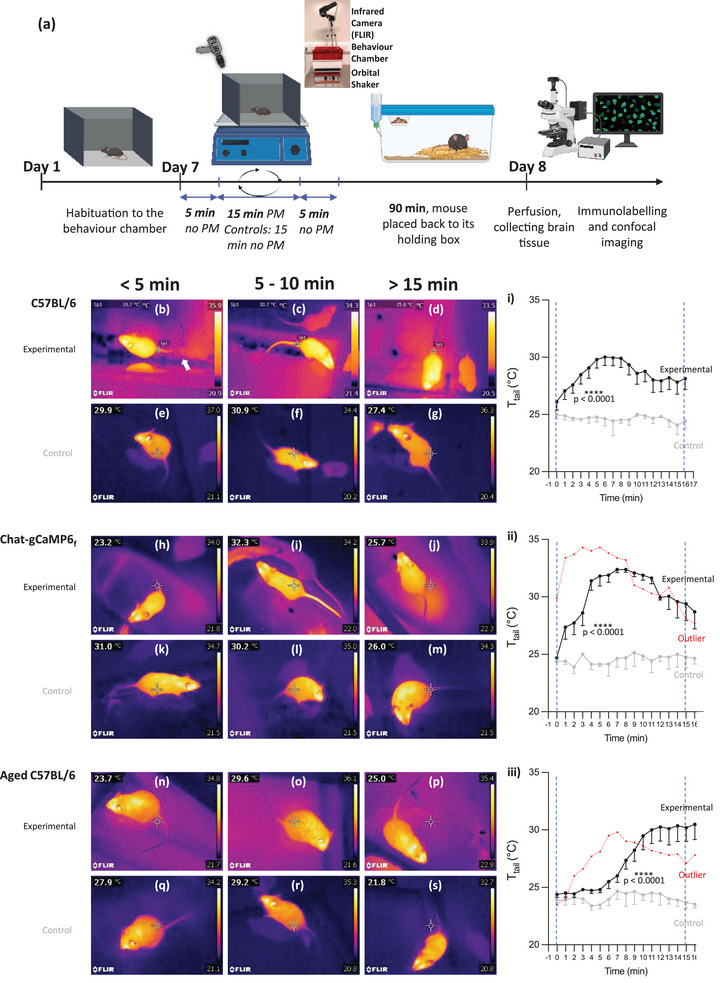
Experimental design and tail temperature (*T*_tail_) response to provocative motion (PM). (a) Experimental design and timeline of experiments; schematic was created by BioRender.com. (b–d; h–j; and n–p) Infrared images of *T*_tail_ responses to PM of C57BL/6; ChAT‐gCaMP6_f_; and aged C57BL/6 mice, respectively. Images were taken during first 5 min of PM; between 5 and 10 min of PM (at peak tail temp), and after 15 min of PM. (e–g; k–m; and q–s) Infrared images of control responses (no PM) of C57BL/6; ChAT‐gCaMP6_f_; and aged C57BL/6 mice, respectively, at the same time intervals as experimental groups. Plot (i) Mean ± *SEM T*_tail_ responses of C57BL/6 experimental group during PM (black trace; *n* = 14) and control (no PM) group (gray trace; *n* = 3). Vertical dashed lines for all plots indicate the start and end point of PM for experimental groups only. Plot (ii) Mean ± *SEM T*_tail_ plots show response of the ChAT‐gCaMP6_f_ experimental group during PM (black trace; *n* = 5) and the *T*_tail_ of the control group PM (gray trace; *n* = 2). An outlier (red dashed line) differed from the mean response to PM (*n* = 1). The outlier trace suggested a summed response of a quicker onset, nonspecific, stress response added to a typical PM stress response. Plot (iii) Mean ± *SEM T*_tail_ plots show response of aged C57BL/6 experimental group during PM (black trace; *n* = 5) and the *T*_tail_ of the control group (gray trace; *n* = 3). An outlier (red dashed line) differed in response to mean PM (*n* = 1) and resembled more closely the young adult C57BL/6 experimental response in Plot i.

Individual experimental mice were placed in the chamber for 5 min before recordings to acclimate them to their surroundings as indicated by baseline *T*_tail_. PM was generated for 15 min at 60 horizontal orbital revolutions per minute. Infrared images were taken every minute beginning just prior to PM onset (0 min) and then every minute during PM (1–15 min) and for 1 min post‐PM (16 min) (Figure 1a). After *T*_tail_ recordings, mice were placed back into their home cage for a further 90 min, a time when c‐Fos expression is expected to peak (Kovács, 2008, 1998). After this waiting period, the mice were transcardially perfused for immunohistochemical labeling (see below).

Control mice were treated similarly. They were habituated to the setup and placed in the recording chamber for the same amount of time as the PM group, and infrared images were also collected every minute. However, control mice were not subject to PM. After 16 min in the recording chamber, control mice were also placed back into their home cage for 90 min before transcardial perfusion for immunohistochemical labeling.

### 2.3 Brain tissue collection and immunohistochemical labeling

Mice were deeply anaesthetized with an intraperitoneal mixture of ketamine (100 mg/kg) and xylazine (0.01 mL/g) and transcardially perfused using heparinized saline, followed by 4% paraformaldehyde (PFA) in 0.1 M phosphate‐buffered saline (PBA). Whole brains were dissected from the skull and postfixed for 3 h in fresh PFA, then rinsed three times for 5 min in PBS, and stored in PBS at 4°C, for a week or less. Prior to sectioning, the brains were cryoprotected by immersion in 30% sucrose/PBS solution overnight, and coronal sections were cut at 50 μm using a Leica CM1950 freezing cryostat.

To label the EVN neurons, brainstem slices of adult and aged‐adult C57BL/6 WT mice were incubated in primary antibodies of anti‐ChAT (1:150) (Merck, ab144P) and anti‐c‐Fos (1:2000) (Abcam, ab190289) for 2 days while continuously rotated on an orbital shaker at room temperature (RT). In Chat‐gCaMP6_f_ mice, brainstem slices were incubated in primary antibodies of anti‐GFP (1:200) (Abcam, ab13970) and anti‐c‐Fos (1:2000) (Abcam, ab190289) for 2 days at RT. Anti‐GFP labeling was used to enhance existing GCaMP signal. After primary antibody incubation, tissue was washed three times in 0.1 M PBS for 10 min and then incubated in secondary antibodies (Alexa 405, 1:50, Abcam ab175665; Alexa 488, 1:200, Abcam, ab150173; and Alexa 594, 1:200, Jackson IR, 711‐585‐152) for 2 h, then washed three times in 0.1 M PBS, mounted, and coverslipped in 50% glycerol/PBS mounting solution. The slices were stored in dark at 4°C until imaging.

### 2.4 Imaging and cell counting

EVN neurons in brainstem sections were imaged using a Nikon C1 confocal microscope with 20×, 40×, and 60× objectives. To count the number of ChAT‐ or GFP‐ and c‐Fos‐positive EVN neurons, the brainstem slices were imaged with a *z*‐stack step size of 0.7 μm. Images were processed and reconstructed as maximum‐intensity projections or three‐dimensional reconstructions using ImageJ (NIH Image) software and EVN neurons were counted. For more details about EVN cell counting, see Lorincz et al. (2022).

### 2.5 Statistics

Statistical analysis was performed in GraphPad Prism software (GraphPad, CA, USA) version 9.4.0. Results of statistical analysis are presented as mean ± *SEM*. To determine the difference of c‐Fos expression between groups, ordinary one‐way ANOVA was used followed by Tukey's multiple comparisons test. Mann–Whitney *U* test was used for nonparametric behavioral *T*_tail_ data. Statistical significance was set at *p* < .05.

## 3 RESULTS

### 3.1 *T*_tail_ response to PM

The average *T*_tail_ of young adult C57BL/6 mice (*n* = 14; Figure 1b–d) was initially 26.11 ± 0.73°C and, after 3 min of PM, increased to 27.06 ± 0.76°C. *T*_tail_ reached a peak within 7 min (30.02 ± 0.74°C) and thereafter began to drop gradually, as previously reported (Tu et al., 2017), to an average temperature of 27.83 ± 1.06°C after 15 min PM (plot [i] in Figure 1). Chat‐gCaMP6_f_ mice (*n* = 5) also exhibited similar pattern of *T*_tail_ responses in response to PM (Figure 1h–j), from a slightly lower average temperature of 24.7 ± 0.3°C at 0 min to 28.6 ± 1.1°C after 3 min of PM, reaching a higher peak temperature of 32.38 ± 0.28°C within 7 min of PM, which then decreased gradually to 29.40 ± 1.48°C at 15 min of PM. In contrast, aged‐C57BL/6 mice (*n* = 5; Figure 1n–p) did not show the typical marked increase in *T*_tail_ response within 7 min of PM (24.38 ± 0.25°C at 0 min, 24.8 ± 0.14°C at 3 min, 25.5 ± 0.61°C at 7 min). Rather, *T*_tail_ only began to rise after 6 min and increased until the end of the recording, reaching an average maximum of 30.2 ± 1.2°C at 15 min of PM.

It should be noted that in both Chat‐gCaMP6_f_ and aged‐C57BL/6 cohorts, there was one outlier that responded differently to their respective cohort. The outlier Chat‐gCaMP6_f_ mouse (plot [ii] in Figure 1, red dashed line) responded more quickly to PM, reaching a peak within 5 min. However, despite the quicker peak *T*_tail_, the decay response of *T*_tail_ was like the rest of the Chat‐gCaMP6_f_ cohort. In the case of the aged‐C57BL/6 outlier (plot [iii] in Figure 1, red dashed line), the response was more like of the younger adult cohorts, reaching a recognizable peak within 7 min. This result may suggest that a limited number of older mice continue to respond to PM in the same way as their younger counterparts. However, to determine how prevalent or what percentage of older animals respond in this way, a population analysis would need to be undertaken, which was beyond the scope of this study design. Despite their responses, the brains of the two outliers were also collected and used for c‐Fos labeling.

The controls of all three cohorts did not show any significant change in *T*_tail_ throughout their time in the recording chamber, as shown by the infrared images of tails of control mice, which remained the same throughout (C57BL/6_control_, Figure 1e–g; Chat‐gCaMP6f_control_, Figure 1k–m; aged‐C57BL/6_control_, Figure 1q–s), and as summarized in adjacent temperature summary graphs (gray plots [i], [ii], and [iii] in Figure 1). Examples of temperatures are as follows: C57BL/6_control_—24.97 ± 0.19°C at 0 min, 24.57 ± 0.07°C at 3 min, 24.63 ± 0.34°C at 6 min, and 24.07 ± 0.48°C at 15 min (*n* = 3); Chat‐gCaMP6f_control_—24.4 ± 0.1°C at 0 min, 25 ± 0.1°C at 3 min, 24.15 ± 0.75°C at 6 min, and 24.65 ± 0.35°C at 15 min (*n* = 2); and aged‐C57BL/6_control_—23.9 ± 0.49°C at 0 min, 24 ± 0.38°C at 3 min, 23.97 ± 0.55°C at 6 min, and 23.70 ± 0.55°C at 15 min (*n* = 3).

All three PM groups showed significantly different *T*_tail_ response compared to controls over the PM period (*U*_C57BL/6 WT_ = 0, *p*_C57BL/6 WT_ < .0001; *U*_Chat‐gCaMP6f_ = 7.5, *p*_Chat‐gCaMP6f_ < .0001; *U*_C57BL/6 aged_ = 12.5, *p*_C57BL/6 aged_ < .0001).

### 3.2 Immunolabeling of EVN neurons and c‐Fos following PM

**FIGURE 2:**
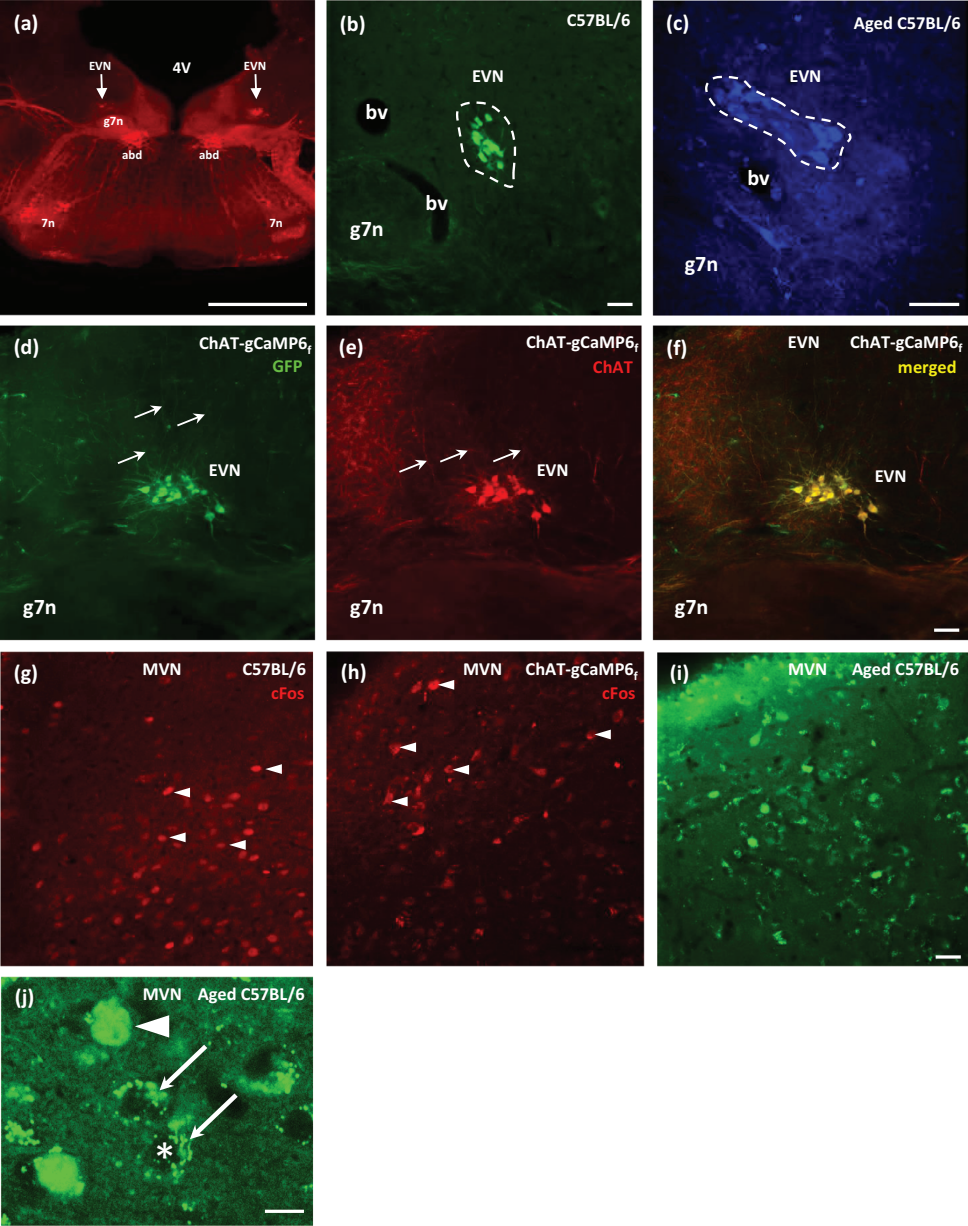
Fluorescent immunolabeling of EVN neurons and c‐Fos protein in the mouse brainstem tissue visualized by confocal microscopy. (a) Low‐magnification image of a C57BL/6 mouse brainstem slice at the level of the EVN. ChAT + Alexa594 (red) was used to label the EVN nuclei bilaterally (arrows) and other cholinergic structures. Scale bar: 1000 μm. (b) Higher power micrograph of ChAT + Alexa488 (green) labeling EVN neurons in C57BL/6 mice. (c) ChAT + Alexa405 (blue) labeling in aged C57BL/6 mice showing fluorescent EVN cells. Areas denoted by dashed lines indicate the tight clustering of EVN neurons dorsal to the genu of the facial nerve (g7n). Double labeling of genetically expressed GCaMP (green, d) and ChAT (red, e) in ChAT‐gCaMP6_f_ transgenic EVN neurons (arrows point to extensive dendritic projections toward the MVN). (f) Merged image of GCaMP and ChAT labeling showing one to one correspondence. (g, h) c‐Fos antibody labeling in the red channel (Alexa594) was verified in the MVN of C57BL/6 and ChAT‐gCaMP6_f_ mice. Arrowheads denote examples of the numerous c‐Fos‐labeled MVN neurons. (i, j) c‐Fos antibody labeling (Alexa488; green) was verified in the MVN of aged C57BL/6 mice. (j) Higher magnification image of aged C57BL/6 mouse showing c‐Fos within the nucleus (arrowhead) and granular appearance of lipofuscin autofluorescence in the cytoplasm (arrows). An asterisk (*) indicates nonlabeled nucleus of lipofuscin labeled cell. Scale bar: 10 μm; (b–i) 50 μm. 4V, fourth ventricle; 7n, facial motor nucleus; Abd, Abducens Nucleus; bv, blood vessel; EVN, efferent vestibular nucleus; gn7, genu of the facial nerve; MVN, medial vestibular nucleus.

**FIGURE 3:**
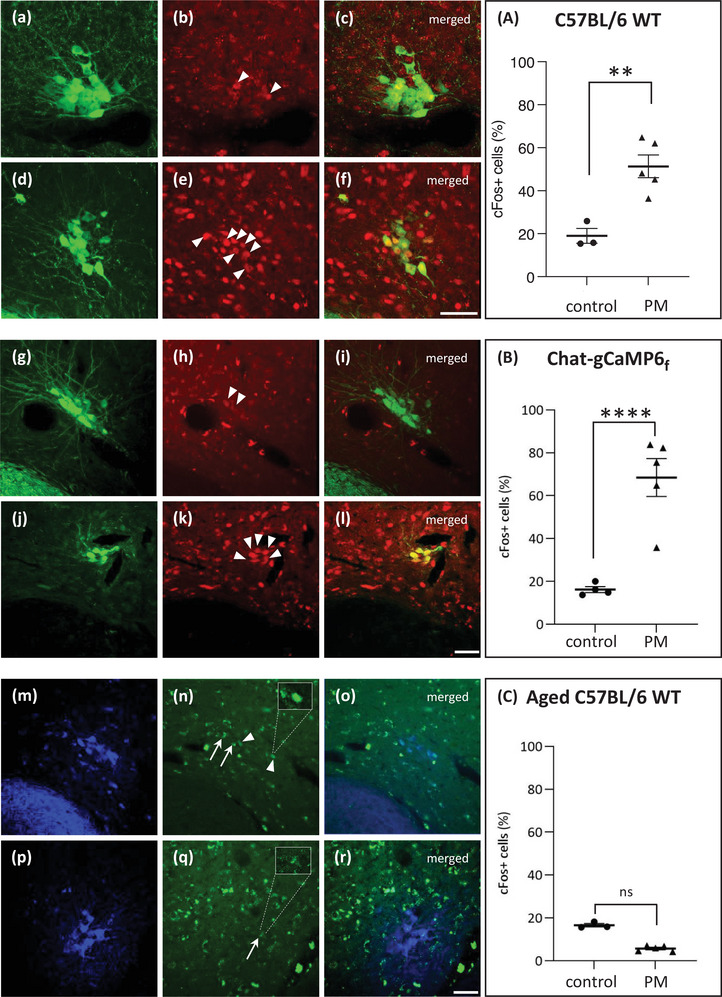
c‐Fos expression in EVN neurons following provocative motion (PM). (a) EVN neurons labeled with ChAT antibody (green) and (b) c‐Fos antibody (red) labeling in the control (no PM) group of C57BL/6 mice. Arrowheads—weakly labeled c‐Fos‐positive EVN neurons. (c) Merged image of panels (a) and (b). (d) ChAT (green) and (e) c‐Fos (red) labeling in the experimental group of C57BL/6 mice after PM. Arrowheads denote c‐Fos‐labeled EVN neurons. (f) Merged image of panels (d) and (e). Graph A shows percentage of c‐Fos‐positive EVN neurons in the control (*n* = 3) and PM group (*n* = 5). **Significant difference between the control and PM group (*p* = .0071). (g) EVN neurons labeled with GFP antibody (green) and (h) c‐Fos (red) labeling in the control group of ChAT‐gCaMP6_f_ mice. Arrowheads—weakly labeled c‐Fos‐positive EVN neurons. (c) Merged image of panels (g) and (h). (j) GFP (green) and (k) c‐Fos (red) labeling in the experimental group of ChAT‐gCaMP6_f_ mice after PM. Arrowheads denote c‐Fos‐labeled EVN neurons. (l) Merged image of panels (j) and (k). Graph B shows percentage of c‐Fos‐positive EVN neurons in the control (*n* = 4) and PM group (*n* = 5). ****Significant difference between the control and PM group (*p* = .0001). (m) EVN neurons labeled with ChAT antibody (blue) and (n) c‐Fos (green) labeling in the control group of aged C57BL/6 mice. Arrowheads denote c‐Fos‐positive EVN neurons enlarged in the inset. Arrows denote lipofuscin autofluorescence. (o) Merged image of panels (m) and (n). (p) ChAT (blue) and (q) c‐Fos (green) labeling in the experimental group of aged C57BL/6 mice after PM. Arrow denotes lipofuscin expressing EVN cell shown in the inset. (r) Merged image of panels (p) and (q). Graph C shows the percentage of c‐Fos‐positive EVN neurons in the control (*n* = 3) and PM group (*n* = 5). Scale bar: 50μm.

The identification, labeling, and counting of EVN neurons were based on our recent study detailing the anatomy of the mouse EVS (Lorincz et al., 2022). Coronal sections (50 μm thick) of PFA‐fixed brains were used for the immunohistochemical detection of c‐Fos and labeling of EVN neurons in the brainstem (Figures 2 and 3). Choline acetyltransferase (ChAT) primary antibody was used to label EVN neurons in C57BL/6 (Figure 2a,b) and aged‐C57BL6 mouse brain tissue (Figure 2c). ChAT immunolabeling proved to be effective regardless of the secondary antibody fluorophore used, for example, red, Alexa 594; green Alexa 488; or blue, Alexa 405 (Figure 2a–c, respectively). Bilateral EVNs in the brainstem were located dorsal and lateral to the genu of the facial nerve (g7n), as previously described in Lorincz et al. (2022). The EVN is easily distinguished from the major cholinergic nucleus in the region, the abducens nucleus (abd), which is located ventral and medial to g7n. The EVN is also significantly smaller in size, has dense cytoarchitecture, and has rich dorsal dendritic projections to the neighboring MVN (Figure 2a).

Presence of the calcium indicator, gCaMP6_f_, in ChAT‐gCaMP6_f_ mouse strain was confirmed and amplified using green fluorescent protein (GFP) immunolabeling (Figure 2d). Counter labeling with ChAT antibody in ChAT‐gCaMP6_f_ mice (Figure 2e) demonstrated that all ChAT‐positive EVN neurons expressed gCaMP6_f_. The two labels (GFP and ChAT) precisely overlapped in this transgenic strain (Figure 2f) and therefore we used GFP labeling in these mice to identify EVN cells.

Given the relatively small number of EVN neurons (mean = 53; Lorincz et al., 2022), c‐Fos antibody labeling was tested in the MVN as a positive control, prior to the EVN experiments (Figure 2g–j). In response to PM, c‐Fos antibody expression with red Alexa 594 secondary fluorescent antibody was observed in the MVN of C57BL/6 (Figure 2g) and ChAT‐gCaMP6_f_ (Figure 2h) mouse strains. No attempt was made to quantify MVN c‐Fos expression. In the aged‐C57BL/6 tissue, c‐Fos antibody labeling together with green Alexa 488 proved to be the most effective labeling combination (Figure 2i,j). However, lipofuscin, a characteristic autofluorescent protein generally found in aged neurons, was also observed in the green channel. Nevertheless, lipofuscin was easily distinguishable from c‐Fos labeling, since c‐Fos gave consistent labeling concentrated around the nucleus of the neurons (see Figure 2j, arrowheads), while lipofuscin appeared as smaller granules deposited at the edges of the soma cytoplasm (see Figure 2j, arrows).

### 3.3 Increased c‐Fos expression after PM in young adult mice but not in aged mice

In C57BL/6 WT mice, cholinergic EVN neurons were labeled using goat anti‐ChAT primary antibody and green Alexa 488 secondary antibody (Figure 3a,d). c‐Fos labeling was visualized using rabbit anti c‐Fos and red Alexa 594 antibodies (Figure 3b,e). Compared to C57BL/6 WT_control_ group (Figure 3a–c), we found strong c‐Fos expression in the C57BL/6 WT_PM_ group (Figure 3d–f). The percentage of ChAT + c‐Fos double‐labeled EVN neurons was significantly higher in the C57BL/6 WT_PM_ group compared to C57BL/6 WT_control_ (*p* = .0071) (Figure 3c,f and Graph A in Figure 3).

In Chat‐gCaMP6_f_ transgenic mice, EVN neurons were labeled using primary antibody against GFP in green (Alexa 488) (Figure 3g,j). Similar to WT mice, c‐Fos was labeled using rabbit anti‐c‐Fos and red Alexa 594 antibodies (Figure 3h,k). As above, significantly higher ChAT + c‐Fos EVN labeling (Graph B in Figure 3; *p* < .0001) was observed in the ChAT‐gCaMP6_f‐PM_ group compared to ChAT‐gCaMP6_f‐control_ group (Figure 3i,l).

In aged C57BL/6 WT mice, EVN neurons were visualized using the blue channel to avoid nonspecific fluorescence from lipofuscin (ChAT and lipofuscin are both localized in the cytoplasm) using goat anti‐ChAT and blue Alexa 405 antibodies (Figure 3m,p). Since c‐Fos labeling was easily distinguishable from lipofuscin, it was labeled green using rabbit anti‐c‐Fos and Alexa 488 antibodies (Figure 3n,q). In the aged cohort, C57BL/6 WT_PM_ did not show significantly higher c‐Fos expression in the EVN compared to their controls C57BL/6 WT_control_ (Figure 3o,r and Graph C in Figure 3; *p* = .7424).

**FIGURE 4:**
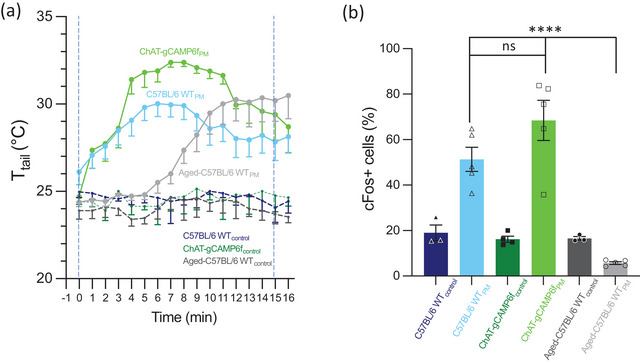
Summary of tail temperature (*T*_tail_) responses to PM and their associated c‐Fos expression. (a) Summary of *T*_tail_ responses to PM of all experimental groups and their controls and (b) the c‐Fos expression in all groups. c‐Fos was significantly different between the young adult C57BL/6 and aged C57BL/6 experimental groups (*p* < .0001) and the ChAT‐gCaMP6_f_ and aged C57BL/6 experimental groups (*p* < .0001). There was no significant difference between young adult C57BL/6 and ChAT‐gCaMP6_f_ experimental groups (*p* = .1730) or aged C57BL/6 control group and aged C57BL/6 experimental group.

Figure 4 shows the summary of c‐Fos labeling in all groups. There was no significant difference between the young adult PM groups: C57BL/6 WT_PM_ and ChAT‐gCaMP6_f‐PM_ (*p* = .1730). As a result, the c‐Fos expression in aged C57BL/6 WT_PM_ mice was significantly less than C57BL/6 WT_PM_ and Chat‐gCaMP6_f‐PM_ (*p*_C57BL/6 WTPM vs aged C57BL/6 WTPM_ < .0001; *p*_ChAT‐gCaMP6f‐PM vs aged C57BL/6 WTPM_ < .0001).

## 4 DISCUSSION

In this study, we present evidence for three findings: (1) increased *T*_tail_ response to provocative horizontal orbital motion in young adult (4–10 months) WT C57BL/6 and transgenic ChAT‐gCaMP6_f_ mouse strains, demonstrating a consistent thermal tail response to motion sickness in mice. (2) Thermal tail response profile significantly altered in aged (>24 months) WT C57BL/6 mice during PM. (3) After PM, we observed increased expression of c‐Fos in EVN neurons of young adult but not in aged mice. Taken together, these results suggest there is raised EVN activity in mice that display signs of motion sickness.

### 4.1 Motion sickness in rodents and PM‐induced thermal response

Our previous study (Tu et al., 2017) reported a typical *T*_tail_ increase during horizontal orbital motion as a sign of motion sickness in CBA mice that peaked within 4–10 min after PM onset and then returned close to baseline of 24–25°C despite continued rotation (Tu et al., 2017). Our findings in a further two mouse strains are consistent with that study and another recent murine motion sickness study (Rahman & Luebke, 2022). In young adult C57BL/6 and transgenic ChAT‐gCaMP6_f_ mice, we found similar *T*_tail_ responses, although ChAT‐gCaMP6_f_ mice showed an elevated peak *T*_tail_ compared to C57BL/6 mice within 6–7 min of PM onset (Figure 4a).

It is unclear why there was a higher *T*_tail_ response in the transgenic strain. Increased blood flow to the extremities, including the tail, can be a result of sympathetic nervous system activation during general stress (Fuller et al., 2010; Jansen et al., 1995). However, the relatively slow time‐to‐peak *T*_tail_ in this strain (6−7 min) suggests it is PM induced for several reasons. First, the contribution of general stress was minimized by prior handling and habituation. Second, during general stress, time‐to‐peak *T*_tail_ occurs more rapidly (1−2 min) compared to PM *T*_tail_ (Rahman & Luebke, 2022). The slower PM time‐to‐peak *T*_tail_ response is thought to be the result of the time taken for sensory conflict to trigger a coordinated program of body cooling. This program includes decreasing body core temperature and thermogenesis in brown adipose tissue while increasing temperature in the extremities, including the tail (Tu et al., 2017) as described in the shrew and rat (Marks et al., 2009; Ngampramuan et al., 2014). In addition to changes in *T*_tail_, other signs of motion sickness were observed in young adult mice. This included cessation of movement and exploratory behavior, tremor, often staying in one corner of the box and lowering their body to the floor of the chamber, and urinary and fecal incontinences (Rahman & Luebke, 2022; Tu et al., 2017; Yu et al., 2007). We made note of these additional behaviors but did not quantify them. By measuring *T*_tail_ and observing behavioral signs, we were able to distinguish motion sickness symptoms from a general stress response. Thus, we interpret the total tail temperature response observed in the “outlier” of plot (ii) in Figure 1 (red plot) as a combination of a fast, initial temperature rise due to an unrelated stress response, as this individual did not show any initial signs of motion sickness during this early period (1–2 min).

Our results also support the idea that aging is a significant factor affecting susceptibility to motion sickness induced by PM in mice. Aged C57BL/6 mice did not show the typical increase in *T*_tail_ peaking at 6–7 min of PM, rather a much slower time course that only began after 6 min (Figure 4a and plot [iii] in Figure 1). Moreover, aged mice did not show the other typical symptoms of motion sickness; they did not stop moving and continued exhibiting exploratory behavior. These results are strikingly similar to observations of an age‐related decrease in motion sickness susceptibility in humans (Lawther & Griffin, 1988; Paillard et al., 2013; Zhang et al., 2016) and rats (McCaffrey & Graham, 1980). In short, the lack of a typical change in *T*_tail_ together with the absence of other motion sickness symptoms in aged mice likely reflects a decreased sensitivity to PM in this cohort.

### 4.2 Increased c‐Fos expression in EVN neurons in response to PM

Previous studies in rats have shown increased c‐Fos protein expression in central vestibular nuclei after vestibular stimulation (Liu et al., 2000, 1999). Specifically, increased c‐Fos expression was observed in the MVN, an important target of afferent vestibular input, after rotational motion (Cai et al., 2010). Moreover, increased c‐Fos expression in the MVN was demonstrated after galvanic vestibular stimulation (Holstein et al., 2012). To date, there have been no studies of c‐Fos expression in vestibular nuclei of mouse or any other animal's EVN following PM. In this study, we found strong expression of c‐Fos after PM in the mouse MVN, consistent with studies in rats, and we also observed increased c‐Fos expression in mouse EVN (Figure 2). It should be noted we only used the MVN as a positive control for c‐Fos immunolabeling (Figure 2g–i), while EVN c‐Fos expression was specifically analyzed (Figures 2a–f and 4b).

We found increased c‐Fos expression in the EVN of young adult WT C57BL/6 and transgenic Chat‐gCaMP6_f_, which suggests increased activation of EVN neurons is related to PM exposure. This would support the assertion there is increased efferent cholinergic activity in the vestibular neuroepithelium as a response to PM, as suggested by Tu et al. (2017).

While PM resulted in increased EVN neuronal activity in young adult mice, there are caveats to c‐Fos as a marker of neuronal activity. The limited temporal resolution of c‐Fos (waiting period of 90–120 min for consequent expression) means it is difficult to determine the exact time course of neuronal activation. For example, does it occur during or after peak *T*_tail_? In addition, c‐Fos also does not provide any information about neuronal connectivity within the nucleus (Kovács, 2008). To fill this gap, further in vivo experiments are needed in live, freely behaving animals to precisely describe EVN neuronal activity before, during, and after PM.

It should be noted that although we identified two outliers in *T*_tail_ measurements (red plots [ii] and [iii] in Figure 1), their tissue was included in c‐Fos processing. Neither outlier showed any difference to their respective c‐Fos cohorts. In the case of the transgenic outlier, the c‐Fos results support the notion that the elevated *T*_tail_ still represented a PM response. In the aged outlier, the unchanged c‐Fos labeling coincides with the absence of other motion sickness signs. However, the *T*_tail_ response was more typical of young adult mice, reaching peak *T*_tail_ 6–7 min after the onset of PM. This discrepancy in the aged outlier, between *T*_tail_ and the lack of increased c‐Fos expression or signs of motion sickness, suggests c‐Fos expression in the EVN may be an even more robust indicator of motion sickness than *T*_tail_.

### 4.3 Effects of aging on the susceptibility of motion sickness

It has been previously reported in humans that gender and age are two major determinants of motion sickness susceptibility; women tend to be more susceptible (Kennedy et al., 1995), while aged individuals seem to be less prone (Paillard et al., 2013; Reason, 1978; Turner, 1999). While sexual dimorphism is of interest, it was not an aim of this study to compare male and female mice since we only had access to males in the aged group. By chance, we had a predominantly female representation in young adult cohorts, but we suggest it should not influence our age‐dependent results significantly since Rahman and Luebke (2022) show there were no differences in PM response between young adult males and females.

The age‐dependent susceptibility in humans appears to peak between 9 and 10 years and begins to decline into middle age and beyond (Cooper et al., 1997; Gahlinger, 2000; Golding, 2006). A similar age dependence occurs in rats (McCaffrey & Graham, 1980; Zhou et al., 2017). Our study of young adult mice (C57BL/6 and transgenic Chat‐gCaMP6_f_) also showed significant sensitivity to PM, while C57BL/6 aged mice did not show the typical elevated *T*_tail_ response.

Aging has destructive effects at different levels of the vestibular sensory system, with reports describing decreased number of (1) vestibular hair cells (Rosenhall, 1973; Rosenhall & Rubin, 1975); (2) Scarpa's ganglion neurons (Richter, 1980) and associated vestibular nerve fibers (Bergström, 1973a, 1973b); (3) neurons in brainstem vestibular nuclei (Lopez et al., 1997); and (4) decreased cholinergic EVN signaling in vestibular hair cells seen in mice (Poppi et al., 2023). All these age‐related changes likely contribute to altered or reduced sensation of balance. In humans, this leads to serious clinical sequelae including falls and injuries (Agrawal et al., 2020). Paradoxically perhaps, it is these same changes that may also result in reduced or absent motion sickness symptoms in response to PM in aged individuals.

Our focus in this study has been the EVS, since we know aging impacts cholinergic systems and can result in significant changes such as cognitive decline in humans (Gallagher & Colombo, 1995). The mechanisms involved include decreasing levels of ChAT, acetylcholinesterase (AChE) (Perry, 1980; Perry et al., 1992), and cholinergic muscarinic receptor binding (Perry, 1980). Moreover, age‐related loss of cholinergic neurons has been reported in the forebrain in humans (Sarter & Bruno, 2004) and rats (Fischer et al., 1992). Similar cholinergic decline has been observed in the inner ear. For example, the number of efferent cholinergic auditory neurons in the gerbil was reduced with age, but perhaps surprisingly, not seen in the EVN (Radtke‐Schuller et al., 2015). Although it was not our main aim to count EVN neurons in this study, we did not see any obvious decrease in EVN neuron number in aged mice, which is consistent with findings in the gerbil. Despite the apparent lack of impact on EVN neuronal numbers in aged rodents, it is still possible that ChAT and AChE and receptor binding levels in the EVS decrease with age, similar to cholinergic changes in other systems.

### 4.4 EVN activation during PM, a possible contribution to nausea symptoms

Vestibular afferents and one of their major central targets, MVN neurons, are reported to project to the EVN (Chi et al., 2007; Li et al., 2005; Liu et al., 2021; Wang et al., 2013). Indeed, the MVN has been shown to have direct excitatory projections (Liu et al., 2021) via ipsilateral glutamatergic (Mathews et al., 2015) closed‐loop circuits to EVN neurons (Chi et al., 2007; Li et al., 2005; Liu et al., 2021; Wang et al., 2013; Zhou et al., 2018). There also appear to be reciprocal connections since EVN neurons provide extensive dendritic branching toward the MVN (Lorincz et al., 2022). Therefore, in young adult mice, PM would produce a significant volley of afferent vestibular input to the MVN that, in turn, would activate EVN cells and therefore increase their c‐Fos expression via glutamatergic short‐loop projections. In contrast, since aging impacts vestibular hair cells and afferent fibers (Bergström, 1973a, 1973b; Richter, 1980; Rosenhall, 1973; Rosenhall & Rubin, 1975), we conjecture that overall vestibular input to the central nervous system is reduced. This reduction would directly impact the MVN/EVN circuitry. Therefore, the stimulus induced by PM would be similarly diminished by aging. The lack of c‐Fos expression in aged mice would support this notion, as do the attenuated motion sickness symptoms, including the significantly altered *T*_tail_ in the first 6–7 min of the PM exposure.

Taken together, our results highlight a potential link between EVS activation and the generation of motion sickness symptoms. For example, elevated activity of the EVN, in response to PM exposure, is strongly associated with symptoms of motion sickness. Similarly, compromised EVS activity corresponds with diminished or the absence of motion sickness symptoms (Tu et al., 2017). Nevertheless, the precise link between EVN neurons and motion sickness symptoms has yet to be determined. The chemoreceptor trigger zone (CTZ) or area postrema is known to control nausea and emesis and is located in the floor of the fourth ventricle of the dorsal medulla oblongata. Its main function is monitoring the cerebrospinal fluid (CSF) and blood for emetic agents and toxins. Since the CTZ and its receptors lie outside the blood–brain barrier, it is able to sample larger molecules within the CSF or those that diffuse out of nearby blood vessels (Borison, 1989). CTZ receptors are sensitive to dopamine, histamine, serotonin, encephalins, substance‐P, acetylcholine (Miller & Leslie, 1994), and PACAP (pituitary adenylate cyclase‐activating polypeptide). Indeed, PACAP‐positive fibers are found within the CTZ (Hannibal, 2002). Although the main fast EVN neurotransmitter is AChE, together with neuropeptides such as calcitonin gene‐related peptide (CGRP) and enkephalins (Perachio & Kevetter, 1989), our preliminary unpublished data show the presence of substance‐P and PACAP in EVN neurons. As we have previously reported, a characteristic anatomical feature of the EVN is its close proximity to a blood vessel, which often bisects it (Lorincz et al., 2022). It is possible that during the EVN activation, some of the neurochemicals mentioned above (especially neuropeptides CGRP, enkephalins, and PACAP) could enter the bloodstream and trigger the CTZ, to generate nausea‐like symptoms and even emesis. A more direct association between the EVN and CTZ has been shown with viral tracing studies that suggest the CTZ provides projections to the EVN, and therefore may influence EVN neuronal activity during the onset of motion sickness (Metts et al., 2006). Understanding the precise link between EVN and nausea and emesis may provide more effective treatment options for motion sickness.

## CONFLICT OF INTEREST STATEMENT

The authors declare no conflicts of interest.

### PEER REVIEW

The peer review history for this article is available at https://publons.com/publon/10.1002/brb3.3064.

## Data Availability

The data that support the findings of this study are available from the corresponding author upon reasonable request.
